# Validity and Reliability of the Dutch Adaptation of the Psoriatic Arthritis Quality of Life (PsAQoL) Questionnaire

**DOI:** 10.1371/journal.pone.0055912

**Published:** 2013-02-13

**Authors:** Freke Wink, Suzanne Arends, Stephen P. McKenna, Pieternella M. Houtman, Elisabeth Brouwer, Anneke Spoorenberg

**Affiliations:** 1 Rheumatology and Clinical Immunology, University of Groningen, University Medical Center Groningen, Groningen, The Netherlands; 2 Rheumatology, Medical Center Leeuwarden, Leeuwarden, The Netherlands; 3 Galen Research Ltd, Manchester, United Kingdom; Keio University School of Medicine, Japan

## Abstract

**Objective:**

The Psoriatic Arthritis Quality of Life (PsAQoL) questionnaire is a disease- specific instrument developed to measure quality of life (QoL) in patients with psoriatic arthritis (PsA). The aim of this study was to translate the measure into Dutch and to determine its psychometric properties.

**Method:**

Translation of the original English PsAQoL into Dutch was performed by bilingual and lay panel. Ten field-test interviews with PsA patients were performed to assess face and content validity. In total, 211 PsA patients were included in a test-retest postal survey to investigate the reliability and construct validity of the Dutch adaptation of the PsAQoL. The PsAQoL, Health Assessment Questionnaire (HAQ) and Skindex-17 were administered on two different occasions approximately two weeks apart.

**Results:**

The Dutch version of the PsAQoL was found to be relevant, understandable and easy to complete in only a few minutes. It correlated as expected with the HAQ (Spearman’s ρ = 0.72) and the 2 subscales of the Skindex-17 (ρ = 0.40 for the psychosocial and ρ = 0.46 for the symptom scale). Furthermore, the measure had good internal consistency (Cronbach’s α = 0.92) and test-retest reliability (ρ = 0.89). The PsAQoL was able to define groups of patients based on self-reported general health status, self-reported severity of PsA and flare of arthritis. Duration of PsA did not influence PsAQoL scores.

**Conclusions:**

The Dutch version of the PsAQoL is a valid and reliable questionnaire suitable for use in clinical or research settings to asses PsA-specific QoL.

## Introduction

Psoriatic arthritis (PsA) is an inflammatory arthritis associated with psoriasis. PsA is characterized by synovitis, dactylitis, enthesitis and spondylitis. The clinical presentation can vary between asymmetrical oligoarthritis, symmetrical polyarthritis or spondyloarthritis. Skin lesions precede the arthritis in most cases. In approximately 15–20% of patients, arthritis is the first presenting symptom (PsA sine psoriasis) [1], [2].

PsA influences the physical and mental status of the patient. Therapy in patients with PsA focuses on both the improvement of symptoms and functioning and on improvement of quality of life (QoL). The most commonly operationalised definition of QoL in health outcomes research is the needs-based model [3]. This model sees QoL as the extent to which a patient is able to meet his or her needs. Symptoms and activity limitations are only important where they prevent needs from being met. QoL is good when most needs are met and poor when disease and/or its treatment prevents need fulfilment. Consequently, QoL is a unidimensional construct, as has been shown by the development of several disease-specific QoL measures [4]–[8].

Different outcome measures have been used to attempt to evaluate the total impact of PsA. Generic health status instruments such as the Health Assessment Questionnaire (HAQ), the Arthritis Impact Measurement Scale (AIMS) and the 36-item Short Form Health Survey (SF-36) are most commonly used. These generic questionnaires predominantly focus on impairment and disability rather than QoL, and their responsiveness has been reported to be limited in PsA [9]. Treatment of PsA with anti-tumour necrosis factor-alpha (TNF-α) blocking agents has been shown to improve QoL in general [10]. The HAQ and SF-36 were used to determine the effect of the intervention, but neither questionnaire actually assesses QoL. To assess changes in outcome associated with interventions accurately, standardized and validated disease-specific instruments are required in PsA. McKenna et al. have developed and validated the PsAQoL questionnaire to fulfil this need [11]. As this instrument was developed with United Kingdom (UK) patients, it requires adaptation for use in other countries. The PsAQoL has recently been adapted for use in several countries for inclusion in international clinical trials but only the Swedish adaptation has been published to date [12]. Prior to the development of the PsAQoL, similar disease-specific questionnaires have been developed and validated for rheumatoid arthritis (RAQoL) [7] and ankylosing spondylitis (ASQoL) [6]. Both questionnaires were developed and validated simultaneously in the Netherlands and the United Kingdom.

The method used to translate all needs-based QoL instruments involves the use of 2 panels, involving native people actually living in the target country. The value of the commonly used translation method – forward and backward translation – has been questioned [13] and there is no evidence supporting its use [14]. In the only head to head study comparing the two panel method with forward and backward translation, the former was shown to produce translations that are more acceptable to patients [15].

The aim of the present study was to translate the English version of the PsAQoL into Dutch and to determine its psychometric properties.

## Methods

Three stages of the Dutch adaptation of the PsAQoL were performed. Stage 1 included the translation of the original PsAQoL, stage 2 the field-testing of the translated version of the PsAQoL and stage 3 the test-retest postal survey of the PsAQoL.

### Stage 1: Translation of the PsAQoL

The two panel methodology was applied to produce the Dutch PsAQoL. This approach has been used in the adaptation of all needs-based QoL instruments [5], [8], [11]. A bilingual panel working together agreed on the best Dutch translations for the instructions and items. The questionnaire was then presented to a lay panel for consideration. This panel was employed to ensure that the language being used in the final version would be understood by potential respondents and that it was in everyday Dutch. This resulted in the first Dutch draft of the PsAQoL.

### Stage 2: Field-testing

Ten interviews were conducted with PsA patients individually to determine face and content validity of the candidate version of the PsAQoL. The interviewer was present during the completion of the questionnaire and determined afterwards whether the interviewee found any problems with the measure, if it was relevant to them and if any important issues were missing. Time taken to complete the PsAQoL was also recorded for each patient.

### Stage 3: Test-retest Postal Validation Survey

#### Patients

289 patients diagnosed with PsA at the outpatient clinic of the Medical Center Leeuwarden were consecutively selected out of the Diagnosis Treatment Combination (DTC)-system from 2009. The DTC-system is a model in which hospitals are paid on a casemix-based tariff for the entire treatment of a patient in the hospital during 1 year. Every DTC, chosen by the medical specialist, has a unique performance code that refers to the clinical diagnosis. Inclusion criteria were fulfilling the Classification criteria of Psoriatic Arthritis (CASPAR) and being over 18 years of age. The selected group of patients contained early as well as established PsA, with early PsA being defined as diagnosed in the previous 2 years and established PsA as diagnosed more than 2 years previously.

All patients received an information letter about the study together with an informed consent form. Patients who did not take the initiative to return the informed consent form were phoned and asked if they wanted to participate in the study. Patients were excluded from the study if no informed consent was given, they were unable to read Dutch or they were legally incapable.

The regional medical ethics committee at Leeuwarden stated that ethics committee approval was not necessary for the study, because the questions on medical information were minimized and used only for calculating the known-groups validity. They concluded that this validation did not fall under the law ‘Medical Scientific Research’.

#### Internal consistency

Internal consistency was assessed by Cronbach’s α coefficient. A value of at least 0.70 indicates adequate inter-relatedness of items [16].

#### Test-retest reliability

The test-retest reliability of an instrument is based on the assumption that the construct has not changed over time and thus the outcome of the measure can be reproduced.

To test the reproducibility of the PsAQoL over time, the questionnaire was completed by the same patients on 2 different occasions approximately 2 weeks apart. This standard time period was chosen because it is unlikely that the disease status will change in this short period which is also long enough to avoid recall of responses. A correlation of 0.85 or above indicates that the measure has good reproducibility [17].

#### Construct validity

The PsAQoL consists of 20 yes/no questions derived directly from qualitative interviews conducted with UK PsAQoL patients [11]. The total score is calculated as the number of questions affirmed and can range from 0 to 20. A high total score indicates a poor quality of life.

Convergent validity of the PsAQoL was evaluated by correlating the PsAQoL scores with those obtained on the HAQ and Skindex-17.

The HAQ measures functional limitations in arthritic disease. The HAQ is a generic instrument that uses a four-point scale to rate performance on 20 tasks. These tasks are grouped in 8 domains: dressing, rising, eating, walking, hygiene, reaching, grip and everyday activities. Score on the HAQ is calculated by adding the highest score of each of the eight domains and dividing by 8. This gives the functional disability index which can range from 0 to 3.

The HAQ focuses on physical aspects of disease and has been shown to be incapable of measuring the impact of skin lesions in PsA [18]. The Skindex-17 questionnaire was included in the survey to capture the impact of psoriasis. Skindex-17 is a Rasch reduced version of the Skindex-29 and consists of 2 subscales (12 psychosocial items and 5 symptom items) [19]. The original 5-point response scale of the Skindex-29 was changed into a 3-point response format. The 2 subscales have separate summing scores, ranging from 0–24 in the psychosocial and from 0–10 in the symptom subscale.

Known-group validity was determined by the degree to which an instrument can demonstrate different scores for groups thought to vary on the factors measured by the scale. Demographic data and information on perceived general health (very good, good, fair or poor), severity of PsA (mild, moderate, severe or very severe) and self-reported flare of arthritis and/or psoriasis during the last 3 months as well as time since diagnosis (early versus established PsA) were collected by a questionnaire and were used to investigate this known group validity.

### Statistical Analysis

Statistical analysis was performed using PASW Statistics 18 (SPSS, Chicago, IL, USA).

The Spearman correlation coefficient was calculated between scores on the 2 administrations of the PsAQoL to determine test-retest reliability. In addition, a Bland-Altman analysis was performed and visualized in a plot to show differences between the first and second assessments of PsAQoL scores.

Construct validity was examined by calculating Spearman’s correlation coefficients (ρ) between the PsAQoL scores and those on the HAQ and on the 2 subscales (psychosocial and symptom scale) of the Skindex-17. It was hypothesized that there would be moderate correlations between scores on these measures as they assess related but different types of outcome. Correlations between 0.5 and 0.7 indicate moderate correlation. The Mann-Whitney U test was used to compare differences between groups. Differences between both assessments were tested using the Wilcoxon signed rank test. P values <0.05 were considered statistically significant.

## Results

Three stages of the validation of the Dutch PsAQoL were performed. First, the original English PsAQoL was translated into Dutch. Second, the field testing of the candidate version of the PsAQoL was performed. And last, the internal consistency, test-retest reliability and construct validity were obtained in a test-retest postal survey.

### Stage 1: Translation of the PsAQoL

The bilingual panel consisted of 3 females and 2 males, aged 24 to 59 years. They worked together on the translation of the original PsAQoL items into Dutch.

The translation of 5 items was straightforward. Fifteen other items raised some discussion (7 items) or could not be translated easily (8 items), because some English expressions do not exist in Dutch or because some English words have more than one Dutch translation. The expert panel changed the answer categories from true/not true to yes/no, because yes and no are more frequently used in Dutch questionnaires.

There were 3 females and 3 males, aged 24 to 62 years in the lay panel, all of whom had an average level of education. The lay panel was asked to review the Dutch questions on their understandability. Unclear words and/or sentences were discussed until consensus was reached. If it was thought to be necessary, changes to words were made without losing the essence of the item.

### Stage 2: Field Testing

Individual interviews were conducted with 5 male and 5 female PsA patients, aged 39 to 79 years. Self-reported severity of PsA and general health ranged from mild to very severe and from poor to very good, respectively. The purpose of the interview was clear to all patients and they found the questionnaire clear and easy to complete. The mean time needed to complete the PsAQoL was 4 (standard deviation (SD) ±2) minutes with a range from 2 to 10 minutes. Median PsAQoL score was 10.5, with a range from 0 to 14.

Five patients suggested missing aspects of QoL in the PsAQoL. Two respondents argued that the influence of PsA on sexual life was important although one of these pointed out that it would be too personal to ask about sexual life in such a questionnaire. Other aspects reported to be missing from the questionnaire were related to impairment and activity limitations rather than QoL or to the opinion of other people regarding their disease.

One respondent stated that it would have been better to use questions rather than statements. All but one patient considered the questionnaire relevant, while another thought that some items were similar. No items were thought to be inappropriate or otherwise unacceptable. Finally, one patient considered some items to be too general.

After reviewing the comments of the interviewed patients with PsA, it was not found necessary to make adjustments to the wording of the Dutch PsAQoL.

### Stage 3: Test-retest Postal Survey

#### Patients

Of the 289 selected patients, 211 (73%) were included in the study. Most of the excluded patients chose not to participate in the study (n = 61), while 15 patients did not respond to the information letter or to several phone calls. One patient was severely mentally impaired and another was excluded because of difficulties with answering questionnaires. The gender (54% male), age (mean 51.9 years) and disease duration (median 6.6 years) of the excluded patients did not differ significantly from that of the included patients.

183 of 211 (86.7%) patients completed and returned the first set of questionnaires. Of these, 145 (79.2%) returned completed questionnaires on the second administration. Characteristics of the validation survey population are shown in Table 1.

**Table 1:** Characteristics of the psoriatic arthritis (PsA) study population.

Number of patients		183^A^	175^B^	134^C^	156^D^
Male gender (n, %)		101 (55)	99 (57)	74 (55)	86 (55)
Age (y) (mean ± SD)		55.4±12.5	55.1±12.7	56.3±12.3	54.9±12.6
Married/cohabitting (n, %)		126 (69)	121 (69)	103 (77)	109 (70)
Employment (n, %)	Full-time	47 (26)	45 (26)	34 (25)	41 (26)
	Part-time	31 (17)	31 (18)	27 (20)	29 (19)
	Retired	31 (17)	28 (16)	26 (19)	24 (15)
	Housekeeper	12 (7)	11 (6)	10 (8)	11 (7)
	Other	29 (16)	28 (16)	26 (19)	25 (16)
	Missing	33 (18)	32 (18)	11 (8)	26 (17)
Duration of PsA (y) (median, range)		5.5 (0.3–45)	5.3 (0.3–45)	5.6 (0.3–45)	5.5 (0.3–45)
Current flare of arthritis (n, %)		27 (15)	25 (14)	24 (18)	23 (15)
Current flare of psoriasis (n, %)		19 (10)	17 (10)	16 (12)	15 (10)
Perceived general health (n, %)	Very good	6 (3)	6 (3)	6 (5)	6 (4)
	Good	71 (39)	68 (39)	56 (42)	64 (41)
	Fair	67 (37)	65 (37)	57 (43)	55 (35)
	Poor	8 (4)	6 (3)	6 (5)	6 (4)
	Missing	31 (17)	30 (17)	9 (7)	25 (16)
Perceived severity of arthritis (n, %)	Mild	62 (34)	58 (33)	47 (35)	55 (35)
	Moderate	65 (36)	67 (38)	58 (43)	59 (38)
	Severe	17 (9)	16 (9)	16 (12)	14 (9)
	Very severe	2 (1)	2 (1)	2 (2)	2 (1)
	Missing	37 (20)	32 (18)	11 (8)	26 (17)
Current treatment for arthritis (n, %)		133 (73)	127 (73)	111 (83)	123 (79)
	DMARD	115 (63)	109 (62)	96 (72)	96 (62)
	Anti-TNFα	27 (15)	25 (14)	21 (16)	22 (14)
	NSAIDs	29 (16)	27 (15)	24 (18)	26 (17)
	Missing	35 (19)	34 (19)	13 (10)	30 (19)
Current treatment for psoriasis (n, %)		57 (31)	53 (30)	45 (34)	46 (30)

Data obtained from self-reports.

A = responders 1^st^ series of questionnaires;

B = 1^st^ series PsAQoL complete; used for internal con- sistency assessment;

C = 1^st^ and 2^nd^ series PsAQoL complete; used for calculating test-retest reliability;

D = 1^st^ series PsAQoL, HAQ and Skindex complete; used for convergent validity analyses.

PsA, psoriatic arthritis; n, number; y, years; SD, standard deviation; DMARD, disease-modifying antirheumatic drugs; anti-TNFα, anti-tumor necrosis factor α; NSAID, non-steroidal anti- inflammatory drug; PsAQoL, psoriatic arthrtitis quality of life; HAQ, health assessement questionnaire.

Thirty patients had early PsA, 119 had established PsA and the time of diagnosis was not known for 34 patients. Of the 133 patients receiving treatment for their PsA, 63% were being treated with Disease Modifying Anti-Rheumatic Drug (DMARD), most frequently being methotrexate and less often salazopyrine, leflunomide or hydroxychloroquine. Fifteen percent of patients were receiving anti-TNF-α treatment.

#### Scores on the outcome measures

Table 2 shows the scores on the PsAQoL, HAQ and Skindex-17 at first and seond assessments. The median PsAQoL score was 5, with a range from 0–20, at both assessments. The scores on the other 2 questionnaires were also relatively low with a wide range.

**Table 2:** Scores on the PsAQoL, HAQ and Skindex-17 at first and second assessment.

	PsAQoL		HAQ		Skin-17 Psychosocial scale		Skin-17 Symptom scale	
	1^st^	2^nd^	1^st^	2^nd^	1^st^	2^nd^	1^st^	2^nd^
N	175	141	170	130	174	143	177	145
Median	5.00	5.00	0.25	0.38	2.00	2.00	4.00	4.00
Range	0–20	0–20	0–3.0	0–2.9	0–24	0–24	0–10	0–10

PsAQoL, psoriatic arthritis quality of life; HAQ, health assessment questionnaire; Skin-17, Skindex-17; N, number of patients.

#### Internal consistency

The Cronbach’s α coefficient for the Dutch version of the PsAQoL was 0.92, indicating adequate inter-relations between the items of the questionnaire (Table 3).

**Table 3:** Internal consistency and test-retest reliability of PsAQoL.

	PsAQoL	
	1^st^ assessment	2^nd^ assessment
Median (range)	5.8 (0–20)	5.8 (0–20)
Cronbach’s α	0.92	0.93
Test-retest reliability(95% CI)	0.89 (0.85–0.92)	

PsAQoL, psoriatic arthritis quality of life; CI, confidence interval.

#### Test-retest reliability

**Figure 1:**
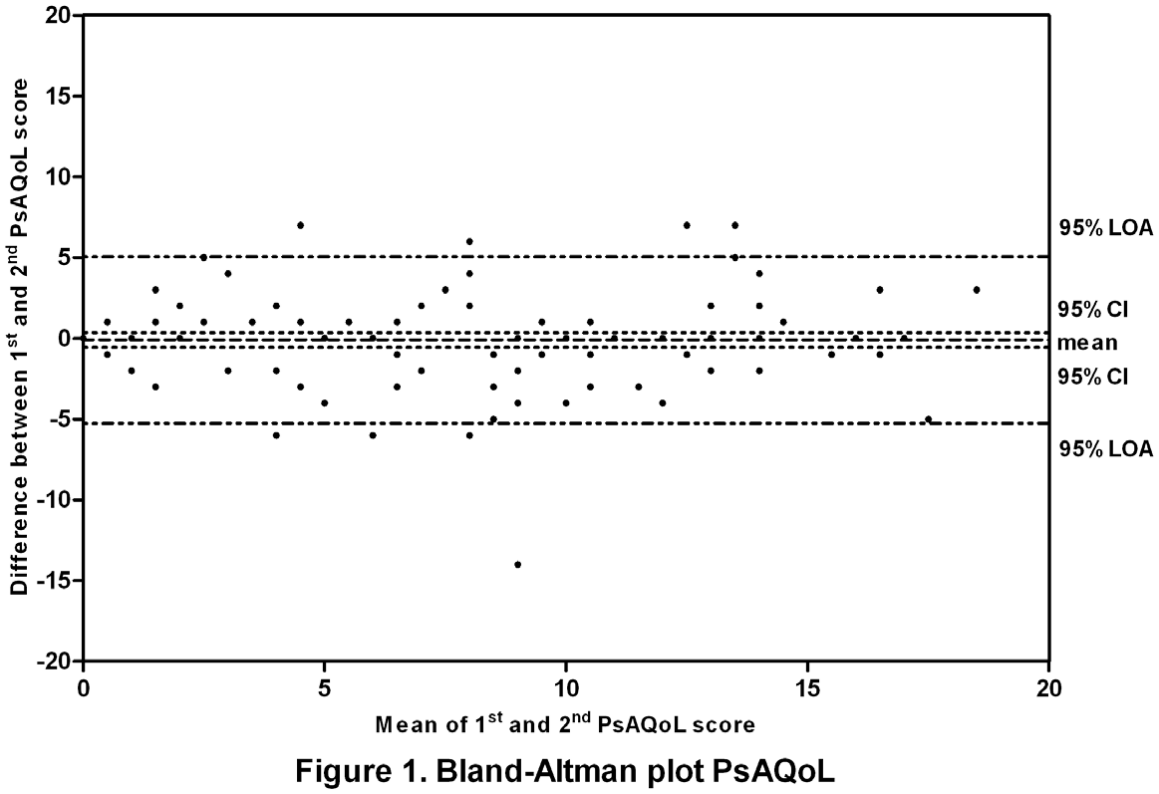
Bland-Altman plot PsAQoL. Difference between the 1^st^ and 2^nd^ PsAQoL plotted against the mean of both assessments. PsAQoL, Psoriatic Arthritis Quality of Life; LOA, limits of agreement; CI, confidence interval.

Test-retest reliability of the Dutch version of the PsAQoL was 0.89, demonstrating very good reproducibility. Bland-Altman analyses showed that the mean difference between the first and second assessment of the PsAQoL was small (except for 1 patient) and did not significantly differ from zero. Consequently, no systematic bias was found between the administrations. Furthermore, the plot was evenly spread around the mean (middle line) (Figure 1). The limits of agreement (LOA) of the PsAQoL were found between -5.3 and 5.1 out of a total of 20.

#### Construct validity

The PsAQoL correlated moderately with the HAQ (ρ = 0.72) and low to moderately with the two subscales of the Skindex-17 (ρ = 0.40 for the psychosocial scale and ρ = 0.46 for the symptom scale) (Table 4).

**Table 4:** Convergent validity of the PsAQoL.

		HAQ	Skin-17 Psychosocial scale	Skin-17 Symptom scale
PsAQoL	1^st^ assessment	0.72*	0.40*	0.46*

PsAQoL, psoriatic arthritis quality of life; HAQ, health assessment questionnaire; Skin-17, Skindex-17.

* P<0.01.

Females scored significantly higher on the PsAQoL than men (median 6.0 vs 2.5, p<0.05). No correlation was found between age and PsAQoL scores was found (ρ = 0.01, p = 0.923).

**Figure 2:**
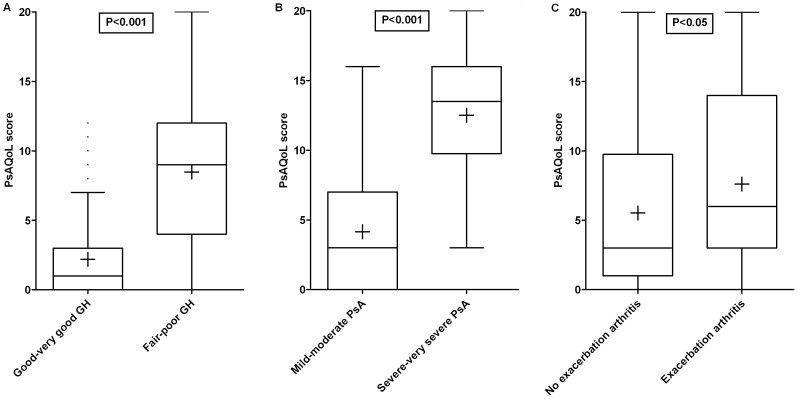
Known-group validity of the PsAQoL visualized in Box-and-Whisker plots (Tukey). Boxes indicate medians with interquartile ranges;+indicate means; whiskers indicate 1.5 times the interquartile distances; • indicate outliers. A = general health, B = severity of psoriatic arthritis, C = exacerbation of arthritis. PsAQoL, Psoriatic Arthritis Quality of Life; GH, general health; PsA, psoriatic arthritis.

Scores on the PsAQoL were related to self-reported general health and severity of PsA and whether or not they were experiencing a flare of their arthritis and/or psoriasis, confirming known-group validity. Patients rating their general health as fair or poor scored significantly higher on the PsAQoL than patients who rated their health good or very good. Respondents perceiving their PsA to be severe or very severe scored significantly higher on the PsAQoL than patients rating their PsA mild or moderate. Patients reporting a flare of their arthritis also had significantly higher scores on the PsAQoL compared to patients without a flare (Figure 2). Time since diagnosis did not significantly influence the PsAQoL scores as was expected (median 5.0 for early PsA vs 3.0 for established PsA; p = 0.537). Changing the definition of early PsA in diagnosed in the previous 5 years did not alter these results (data not shown). No statistically significant differences in PsAQoL score were found for patients reporting a flare of psoriasis compared to patients not reporting a flare (median 6.5 vs 3.0, p = 0.122).

## Discussion

The Dutch version of the PsAQoL was found to be relevant, understandable and easy to complete in only a few minutes. It showed good internal consistency, reproducibility and construct validity.

The good internal consistency of the PsAQoL found in the present study is in line with the findings of the original development study of the PsAQoL and the adaptation study of the Swedish PsAQoL [11], [12]. Also, reproducibility was found to be good in all 3 studies. The moderate correlation of the PsAQoL to the HAQ and Skindex-17 reflect the fact that these measures only assess one facet of PsA and that they measure different types of patient-reported outcome.

The PsAQoL was able to distinguish patients perceiving worse self-reported general health or more severe PsA. Also patients experiencing an exacerbation of arthritis could be discriminated. The duration of the PsA did not affect the score on the PsAQoL. These results are in accordance with the studies in the UK and Sweden [3], [12]. Both studies also found differences in PsAQoL score based on (self-reported) general health and exacerbation of arthritis. Perceived severity of PsA was related to the PsAQoL score in the original PsAQoL study [3]. Both studies did not demonstrate differences in score related to time since diagnosis of the PsA.

PsAQoL scores in this study were low compared to scores in the PsAQoL developmental study reported by McKenna et al [11], but similar to those found in the Swedish study [12]. The median in the present study was 5 versus 9 in the original publication and 5.8 in the Swedish study. In both adaptation studies, approximately 20% of the patients scored the minimum whereas only 1% scored the maximum. The low scores on the PsAQoL in this study probably reflect the relatively mild disease in the sample studied. In the present study, 72% of the patients reported their PsA as being mild or moderate and 42% rated their general health to be good or very good. In the UK study, 63% of the patients classified their arthritis as mild or moderate. The numbers of flares of arthritis and psoriasis was low in this study (15% and 10%) compared to both the original PsAQoL study (54% and 41%) and the Swedish study (47% and 45%) [11], [12]. Treatment for arthritis was given in 73% of patients in both this and the UK PsAQoL validation population. The difference in the severity of PsA in the Swedish and present study compared to the UK study might be partly explained by the increase in use of biological treatment in PsA.

In the OMERACT 8 (Outcome Measures in Rheumatoid Arthritis Clinical Trials), a PsA module was included which led to a core set of 6 domains, one of these being health-related QoL [20]. While the PsAQoL assesses QoL rather than health-related QoL, it has the advantage that the needs-based measurement model copes well with the presence of both skin and joint related problems.

Sensitivity to change is an important requirement for outcome measures. To determine whether the PsAQoL could detect significant change over time Healy and Helliwell [21] analysed the responsiveness to change of the PsAQoL in a group of 28 patients with active PsA. They found a standardized response mean (SRM) of 0.71 at 3 months and of 0.41 at 6 months. Similar rates of change for Patient Global VAS and the HAQ were found (which have both been validated for PsA). Before the PsAQoL can be used in intervention studies, further research is necessary to evaluate the sensitivity to change of the PsAQoL.

Marzo-Ortega et al. [22] published a small clinical trial in which they used the PsAQoL as a secondary outcome measure. At 20 weeks there was a significant improvement in PsAQoL scores for patients with active PsA treated with infliximab and methotrexate.

In conclusion, the present study showed that the Dutch version of the PsAQoL is valid and reliable. Patients found the questionnaire relevant, understandable and easy to administer, taking about 4 minutes to complete. These results suggest that the PsAQoL can be a relevant questionnaire for use with PsA patients in Dutch clinical and research settings.
